# Supplementary material for: Assessing the ecological resilience of Ebola virus in Africa and potential influencing factors based on a synthesized model
Source: PLoS Negl Trop Dis. 2025 Feb 7;19(2):e0012843. doi: 10.1371/journal.pntd.0012843 (PMC11805440; doi:10.1371/journal.pntd.0012843)
Supplement: S1 Appendix — Fig A. Multiple data sources, including Ebola epidemic case data, surveyed attribute data, remote sensing data, and spatial vector boundaries, and establishment of a geodatabase. The base layers of the map were obtained from the openly available source via the Natural Earth (https://www.naturalearthdata.com/downloads/50m-cultural-vectors/). Fig B. Heat map of spearman coefficients for each variable illustrating the correlations among variables. Table A. The advantages and disadvantages of Maxent, Bioclim, Domain, and GARP models. (DOCX) [file pntd.0012843.s001.docx]

***Appendix A***

**Assessing the ecological resilience of Ebola virus in Africa and potential influencing factors based on a synthesized model**

**Figure of Contents**

| **Fig A** | - Figure S1. Multiple data sources, including Ebola epidemic case data, surveyed attribute data, remote sensing data, and spatial vector boundaries, and establishment of a geodatabase. The base layers of the map were obtained from the openly available source via the Natural Earth (https://www.naturalearthdata.com/downloads/50m-cultural-vectors/). |
| --- | --- |
| **Fig B** | Heat map of spearman coefficients for each variable illustrating the correlations among variables. |

**Table of contents**

| **Table A** | The advantages and disadvantages of Maxent, Bioclim, Domain, and GARP models |
| --- | --- |

**
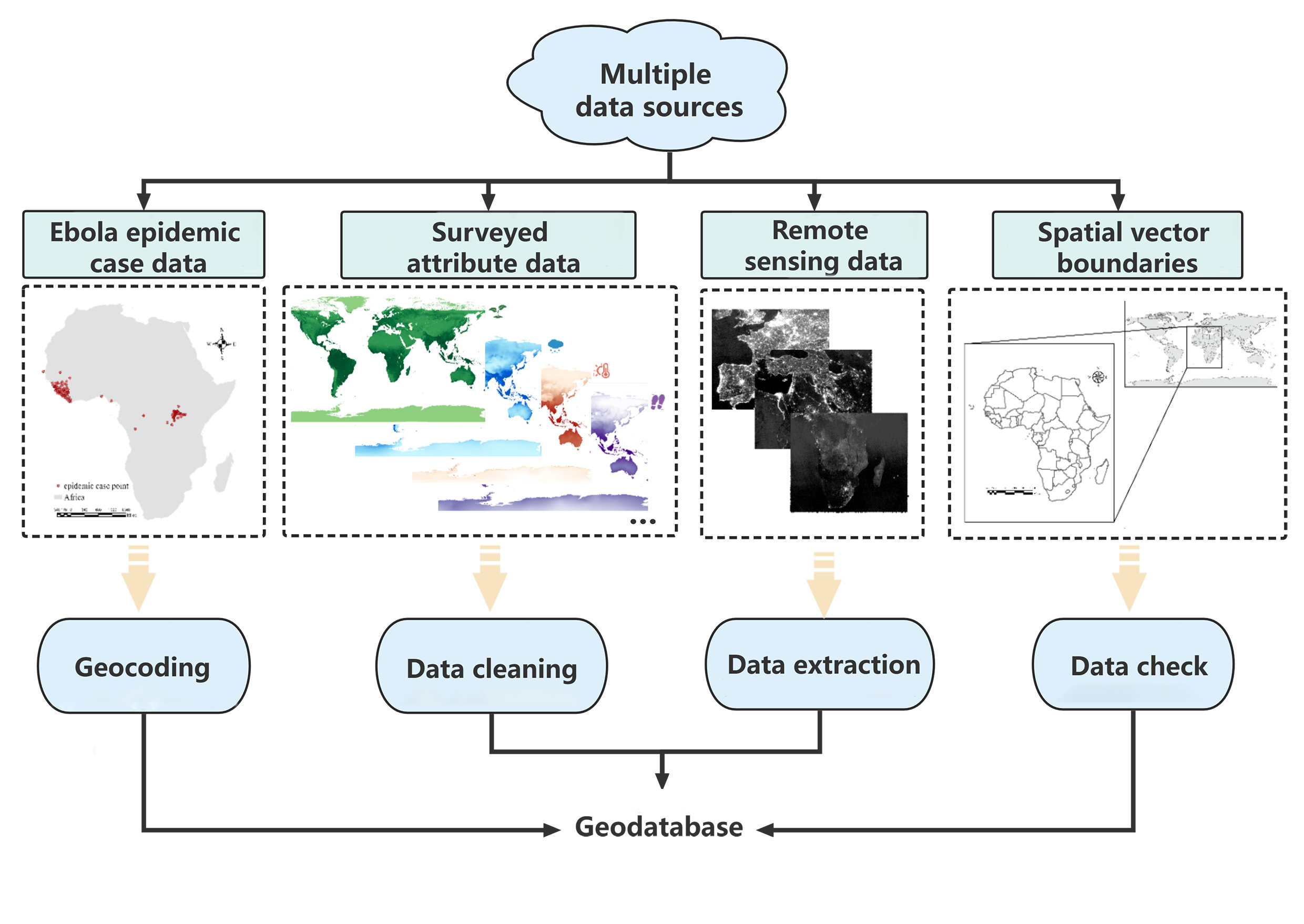
**

**Figure A.** Multiple data sources, including Ebola epidemic case data, surveyed attribute data, remote sensing data, and spatial vector boundaries, and establishment of a geodatabase. The base layers of the map were obtained from the openly available source via the Natural Earth (https://www.naturalearthdata.com/downloads/50m-cultural-vectors/).

**
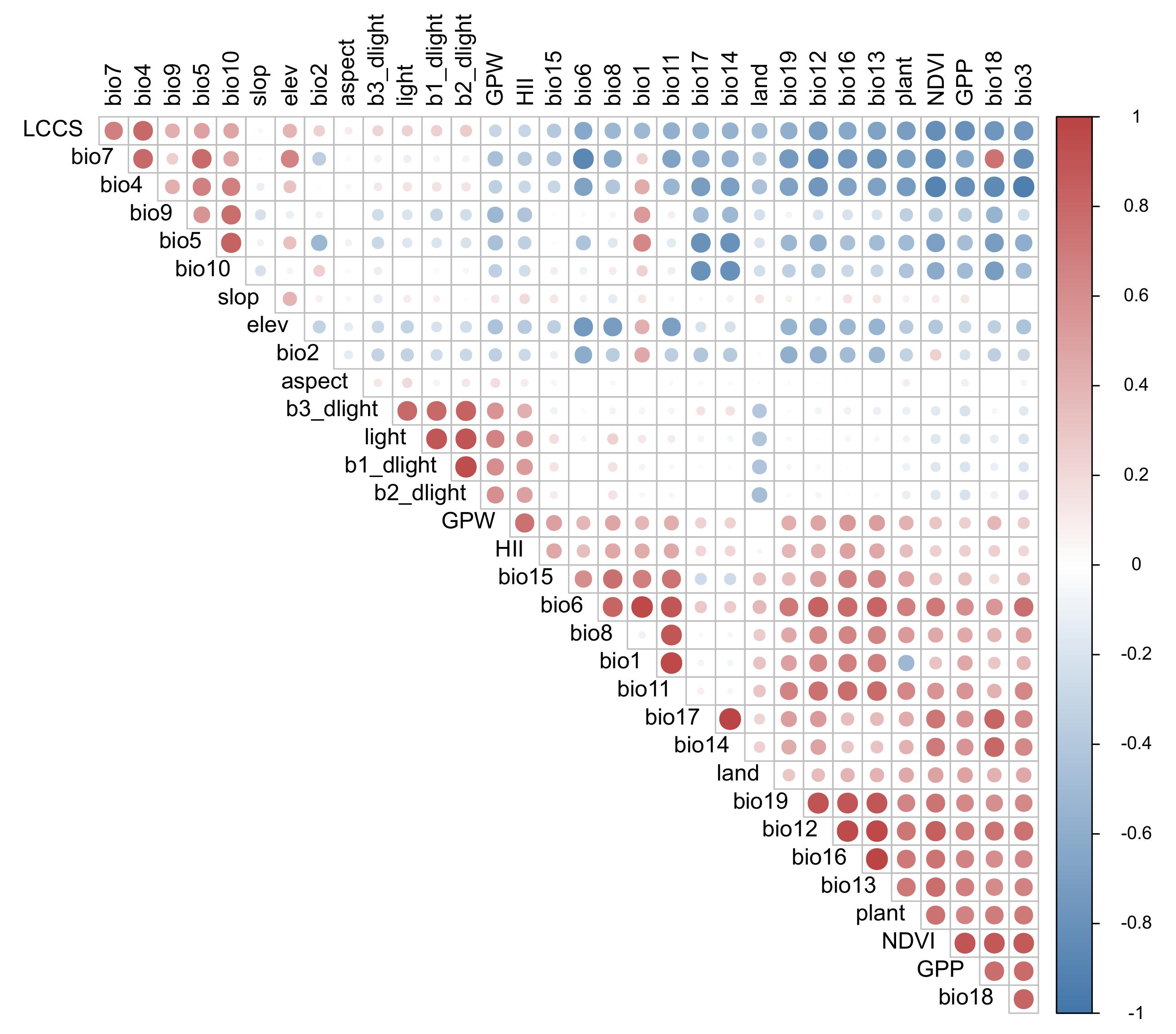
**

**Figure B.** Heat map of spearman coefficients for each variable illustrating the correlations among variables.

**Table A.** The advantages and disadvantages of Maxent, Bioclim, Domain, and GARP models

| **Model** | **Advantages** | **Disadvantages** |
| --- | --- | --- |
| Maxent [31] | - Efficiently handles sparse data and complex interactions - Suitable for high-dimensional and nonlinear data - Strong regularization prevents overfitting | - Numerous parameters, complex model adjustment - Requires substantial data for optimal performance |
| Bioclim [30] | - Simple operation, easy to understand and implement - Stable predictions based on extreme climate values | - Ignores non-climatic ecological factors - Unable to identify interactions among climatic variables |
| Domain [32] | - Intuitively reflects species-environment similarity - Suitable for data-limited rare species | - Sensitive to outlier data, easily disturbed - Simplified models may overlook important environmental variables |
| GARP [33] | - Iterative optimization enhances model performance - Effectively handles complex non-linear rules | - High computational and resource demands - Results may vary significantly |
