# Supplementary material for: Assessing the ecological resilience of Ebola virus in Africa and potential influencing factors based on a synthesized model
Source: PLoS Negl Trop Dis. 2025 Feb 7;19(2):e0012843. doi: 10.1371/journal.pntd.0012843 (PMC11805440; doi:10.1371/journal.pntd.0012843)
Supplement: S2 Appendix — Table A. The format requirements of the input data for models used in this study. (DOCX) [file pntd.0012843.s002.docx]

***Appendix B***

**Assessing the ecological resilience of Ebola virus in Africa and potential influencing factors based on a synthesized model**

Characteristics and introduction of the models used in this study

**Maxent model**

The Maxent model is grounded in the principle of maximum entropy, leveraging species occurrence data in combination with known influential factors to create sample points. Maxent's versatility enables the fitting of intricate functions, facilitating non-linear modeling with high-dimensional data. Moreover, the model has advantages in capturing interaction effects and effectively addresses overfitting concerns through impressive regularization prowess. In the context of Ebola, the ability to incorporate diverse environmental and socio-economic variables makes Maxent particularly valuable for modeling the complex dynamics of outbreaks across varied geographical regions. However, it requires substantial data input to optimize its predictive accuracy, which might be a limitation in data-scarce regions.

In mathematical terms, the Maxent model optimizes the conditional entropy, denoted by $H\left( y|x \right)$, where $y$ represents the species occurrence and $x$ denotes the influential factors within the training dataset $\mathbf{x}$ . The optimization is achieved by minimizing the negative sum of joint probabilities $p\left( y,x \right)$ multiplied by the logarithm of the conditional probabilities $p\left( y|x \right)$, which is modeled as:

$$\begin{aligned} H\left( y \mid x \right)=-\sum_{\left( x,y \right)\in\mathbf{x}} p\left( y,x \right)\log p\left( y \mid x \right)\#\left( 1 \right) \end{aligned}$$

The model seeks the optimal probability distribution $p^{*}\left( y|x \right)$ by maximizing $H\left( y|x \right)$ under the constraint that the distribution belongs to the set $P$. This constraint is expressed as:

$$\begin{aligned} p^{*}\left( y|x \right)=arg\max_{p\left( y|x \right)\in P}H\left( y|x \right)\#\left（ 2 \right） \end{aligned}$$

**Bioclim model**

The Bioclim model primarily compares characteristic of the study area with that of the bioclimatic envelope to explore the potential non-random relationships. It utilizes the Percentile Distribution algorithm to perform multivariate one-sided analysis on each independent variable of every grid. If all variables of a particular area fall within the bioclimatic envelope of the species, then that location can be considered suitable for species survival. For Ebola, while Bioclim can help identify climatic conditions favorable for the virus or vector habitats, its effectiveness may be reduced by not considering non-climatic factors like population contact and land use, which are crucial in the spread of the virus. The Bioclim model provides a rigorous and logically consistent approach to assess the ecological suitability for species within a given area.

**Domain model**

The Domain model predicts the distribution area of a species by comparing the independent variables of the focal point with those of the surrounding areas using the Gower matrix. It measures the similarity between the influential variables to estimate the resemblance to the known distribution areas.The maximum similarity value is 100, with higher values indicating greater similarity to the known distribution areas. For Ebola, this model's strength in assessing environmental similarity can be used to predict new outbreak zones based on ecological matches; however, its focus on environmental factors alone might overlook the impact of human behavior on outbreak dynamics.

In specific terms, the Domain procedure employs a point-to-point similarity metric to attribute a classification value to a candidate site based on its proximity in environmental space to the most similar record site. The Euclidean distance $d$ between two points A and B in a p-dimensional space is defined as:

$$\begin{aligned} d_{\mathrm{AB}}=\frac{1}{p}\sum_{k=1}^{p} \left( \frac{\left| A_{k}-B_{k} \right|}{\mathrm{range}k} \right)\#\left( 3 \right) \end{aligned}$$

The complementary similarity measure $R_{\mathrm{AB}}$ is expressed as:

$$\begin{aligned} R_{\mathrm{AB}}=1-d_{\mathrm{AB}}\#\left( 4 \right) \end{aligned}$$

The maximum similarity $S_{A}$ between candidate point A and the collection of known record sites $T_{m}$ is articulated as:

$$\begin{aligned} S_{A}=\overset{m}{\max_{j=1}} R_{AT_{j}}\#\left（ 5 \right） \end{aligned}$$

Assessing $S$ for every grid point within a designated region results in a matrix of continuously changing similarity values, which can be used for subsequent niche prediction.

**GARP model**

The Genetic Algorithm Rule-set Production (GARP) model is characterized by a rule-based approach. Its fundamental operating principle involves an iterative process where four rule-sets are selected as genes and passed down through generations using genetic algorithms. In each generation, an attempt is made to incorporate a new rule-set, which is then tested to determine if the change can bring positive gains. Rule-sets that contribute to gains are retained, resulting in a complete model composed of multiple rule-sets. The four rule-sets are as follows: atomic rule-set, bioclimatic envelope, logistic regression, and inverse bioclimatic envelope. While GARP's iterative nature allows it to adapt well to incorporating new data, it tends to be more generalized and may not sufficiently consider specific ecological characteristics, which could limit its accuracy in predicting complex ecological dynamics such as those seen with Ebola outbreaks.

**GAM**

The principle of the generalized additive model involves non-parametric regression by summing the predictor variables to compute a non-parametric function of the relationship between predictor variables and response variables. Unlike typical additive models that assume the response variable follows a normal distribution, the variables employed in this study, theoretically speaking, do not adhere to a normal distribution. Hence, a generalized additive model is utilized. The specific formula is presented below:

$$\begin{aligned} Y=\beta_{0}+f_{1}\left( X_{1} \right)+f_{2}\left( X_{2} \right)+\cdots+f_{n}\left( X_{n} \right)\#\left（ 6 \right） \end{aligned}$$

Here, each $f_{i}(\cdot)$ denotes a non-linear smooth function capturing the relationship with the corresponding predictor variable. The error term $\beta_{0}$ accounts for unobserved factors contributing to random variability in the model.

Specifically, in the experimental procedure, random points were generated globally. Environmental data corresponding to these points, along with Maxent model outcomes, were extracted. The model results were then employed as the response variable, fitting them against variations in environmental data. The aim was to observe the response of the model outcomes to changes in environmental variables.

**Model building method**

For the Maxent model, we employed Maxent software V3.4.3, a Java-based tool designed by Steven J. Phillips from Columbia University for predicting the potential distribution of species. We utilized the R package ENMeval for tuning parameters, which allows running ecological niche models across all combinations of user-defined settings and performs cross-validation to evaluate the models. For model training, we selected optimal parameters, using the following settings: Feature classes (fc) set to LQ (Linear and Quadratic features), and a regularization multiplier (rm) of 2, which controls the regularization strength, with higher values yielding broader distributions.

For the Bioclim and Domain models, we based the analysis on DIVA-GIS software. First, we imported the case point data and the prediction range in vector format. Then, we used the DIVA-GIS functionality to merge the environmental raster data into one stack file, based on which we trained and analyzed the models. The models were executed using the default settings of the DIVA-GIS software. For specific parameter details, readers can refer to section 7.1 of the DIVA-GIS user manual (https://diva-gis.org/docs/DIVA-GIS_manual_7.pdf).

For the GARP model, we used DesktopGARP 1.1.3 software. Initially, we converted tif raster files from the database into asc files, created a mask file based on the scope, rows, columns, and pixel size of the raster files, and then converted the asc files into raw files. Subsequently, we input those files along with the case point vector data into the software for model training. The following parameters were used in DesktopGARP: Runs = 20, Convergence Limit = 0.01, Max Iterations = 1000, Models Under Hard Threshold = 20, and Commission Threshold = 50. A tutorial video on DesktopGARP parameter settings is available at <https://www.youtube.com/watch?v=vIzRiXA2aOE.>

For all four models above, we used the same primary datasets: species occurrence data, provided as a .txt file containing latitude and longitude coordinates of cases, and environmental variable data, provided in .tif format as continuous raster data. While the input datasets are consistent across models, each model has specific requirements for data formatting. A summary of the input data requirements is provided in Table S2 to aid reproducibility.

**Table A.** The format requirements of the input data for models used in this study.

| Model | Case distribution points | Variable |
| --- | --- | --- |
| Maxent | .txt | .asc |
| Boclim/Domain | .shp | .grid |
| GARP | .xls | .asc |

For the GAM, we employ R programming to generate random points globally. We then extract the corresponding environmental data for these points, as well as results from the Maxent model. These results are used as response variables input into the GAM, which allows us to fit and observe the response of the model outcomes to changes in environmental variables.
