# Supplementary material for: Assessing the ecological resilience of Ebola virus in Africa and potential influencing factors based on a synthesized model
Source: PLoS Negl Trop Dis. 2025 Feb 7;19(2):e0012843. doi: 10.1371/journal.pntd.0012843 (PMC11805440; doi:10.1371/journal.pntd.0012843)
Supplement: Attachment — Submitted filename: Response to Reviewers - 03.docx [file pntd.0012843.s004.docx]

10-Aug-2024

Dear Editor,

Thank you so much for your kind consideration of our manuscript entitled ***‘Assessing the ecological resilience of Ebola virus in Africa and potential influencing factors based on a synthesized model****’* (Manuscript reference number: **PNTD-D-24-00256**) and for sharing the insightful review comments which have helped improving our manuscript a lot. We have carefully considered all the comments and suggestions from the two reviewers and revised the manuscript accordingly. Enclosed please find our point-by-point responses to their comments and suggestions. All the corrections have been highlighted in yellow of the revised manuscript. We hope you will find our manuscript appropriated for publication in ***PLOS Neglected Tropical Diseases.***

As before, this manuscript is not under consideration for publication by any other journal or medium, and all authors have agreed to its publication.

We look forward to your reply.

Sincerely yours,

Kun Liu, PhD

Department of Epidemiology, School of Public Health,

Air Force Medical University,

169 Chang-Le Street, Xincheng District, Xi’an 710032, People’s Republic of China,

Tel: +86-015691759599

Email: liukun5959@qq.com

**<Methods>**
**Reviewer #1: None
Reviewer #2:**

1. **The objectives are clearly stated, however the methodology and data are not clearly explained.**

**Thank you for your valuable feedback. We have carefully considered your comments and have thoroughly revised the methodology and data sections of our manuscript to ensure clarity and comprehensiveness.**

2. **Line 108: The final number of cases and deaths according to WHO was 3481 and 2291 respectively. Please update and provide a citation.**

**Thank you for pointing out this problem. We have updated those numbers which should be 3481 and 2299 for current citation as follows (Page 11, line 110):**

*WHO (World Health Organization). 2021. Ebola outbreak in Democratic Republic of the Congo declared over. Available from: https://www.afro.who.int/news/ebola-outbreak-democratic-republic-congo-declared-over. Accessed May 03, 2021.*

3. **Line 112: Please provide a citation to support this statement: I find it very surprisingly that there would be an established epidemiological link between the 2014 West African outbreak and the 2022 Uganda outbreak given that the former was the Zaire subtype whilst the latter was the Sudan subtype.**
(1) **Thanks for your valuable comments. We can understand your concern and are aware of the inappropriateness and inaccuracy of this statement. Our original intention was to emphasize the focus on Ebola outbreaks occurring after 2014. Therefore, to avoid any misunderstanding, we have revised the statement into a clear one *Due to the sporadic nature of Ebola outbreaks prior to 2014, we focused our investigation on the ecological adaptability of the EVD based on outbreaks occurring after the 2014 West Africa epidemic* (Page 11, lines 113-115)*.***
(2) **In addition, we added a citation [22] here:** *Weyer J, Grobbelaar A, Blumberg L. Ebola virus disease: history, epidemiology and outbreaks. Current infectious disease reports. 2015;17(5):480.* **In this paper the statement *Before 2014, EVD outbreaks have been characterized in their occurrence only by their random nature, occurring after periods of apparent epidemiological silence and only rarely reoccurring in the same location can* support this point.**
(3) ***Furthermore, addressing the issue of Ebola virus species, due to the small data size of available Ebola cases categorized by Ebola virus species, our study did not conduct the analysis based on different types of Ebola viruses. We have addressed this limitation in the discussion section*(Page 28, lines 427-431)*.***
4. **Line 120: Please provide a link and citation to the actual dataset used, not just the platform. What variables did you extract: case numbers, deaths, or death? What temporal resolution (if any) did you use?**

**Thanks very much for pointing out this problem. According to the reviewer’s suggestion, we have changed the HDF platform into a link to a specific dataset. The 2018 DRC outbreak comes from** <https://data.humdata.org/dataset/drc-health-facilities.> **The 2014-2016 outbreaks are sourced from** <https://data.humdata.org/dataset/evd-cases-by-district.> **And we mainly extracted the case numbers from the dataset with a daily temporal resolution, which has been added in the text (Page 11, lines 119-120).**

5. **Line 126: Again, please provide a link to the exact data source, not just the data platform.**

**Thanks again. Since the information of the 2022 outbreak in Uganda was directly sourced from the Global Health homepage, we provided the link to this data source as follows:** https://global.health/ **(Page 12, line 121)**

6. **Line 136: This is not the correct reference.**

**Thank you very much for your thorough review of the manuscript. This is our mistake that the reference was misplaced due to a numbering error when we organized the citations (Page 12, line 138). We sincerely apologize for this mistake and have replaced it with the correct one [23] as follows:**

*Fang LQ, Yang Y, Jiang JF, Yao HW, Kargbo D, Li XL, Jiang BG, Kargbo B, Tong YG, Wang YW, Liu K. Transmission dynamics of Ebola virus disease and intervention effectiveness in Sierra Leone. Proceedings of the National Academy of Sciences. 2016 Apr 19;113(16):4488-4493.*

7. **Line 137: Please link to the exact database and provide more details on the variable used, their meanings and the resolution of the data. Please do the same for the CCCS, NDVI and AVHRR data. Did you consider any variables related to bat habitats, given they are a reservoir for Ebola?**
(1) **We really appreciate the reviewer’s constructive comments. According to the reviewer’s suggestion, we have supplemented data description in the original table of the appendix with links to the specific datasets and more details on all the variables used and mentioned by the reviewer, including their meanings and resolutions. The final spatial resolution of our data is 2.45 km after data processing. To make it more clear and understandable, we have moved this table from the appendix into the main text as Table 1 (Page 19) as follows:**

**Table 1.** Description of the selected 13 variables for this study, including their units, ranges, STD, means, and data sources.

| **Variables** | **Minimum** | **Maximum** | **Mean** | **STD** | **Source** |
| --- | --- | --- | --- | --- | --- |
| Isothermality (bio3) | 9.1 | 100.0 | 34.4 | 18.8 | https://www.worldclim.org/data/worldclim21.html |
| Temperature Seasonality (×100; bio4) | 0 | 2377.6 | 888.5 | 467.8 |  |
| Min Temperature of Coldest Month (℃; bio6) | -72.5 | 26.5 | -20.2 | 26.0 |  |
| Temperature Annual Range (℃; bio7) | 1 | 72.7 | 34.1 | 12.0 |  |
| Mean Temperature of Coldest Quarter (℃; bio11) | -66.4 | 29.3 | -14.3 | 26.7 |  |
| Annual Precipitation (mm; bio12) | 0 | 11246.0 | 537.2 | 636.5 |  |
| Precipitation of Driest Month(mm; bio14) | 0 | 507.0 | 14.7 | 27.6 |  |
| Gross Primary Productivity(GPP) | 0 | 715.2 | 44.2 | 85.7 | https://essd.copernicus.org/articles/14/1063/2022/essd-14-1063-2022-assets.html |
| Gridded Population of the World(GPW) | 0 | 357341.3 | 46.2 | 423.7 | https://sedac.ciesin.columbia.edu/data/set/gpw-v4-population-count-rev11 |
| Human Influence Index(HII) | 0 | 64 | - | - | https://sedac.ciesin.columbia.edu/data/set/wildareas-v2-human-influence-index-geographic |
| Land Cover Classification System(LCCS) | 10 | 220 | - | - | https://maps.elie.ucl.ac.be/CCI/viewer/ |
| Night Lights(light) | 0 | 255.0 | 4.1 | 20.0 | https://eogdata.mines.edu/products/dmsp/ |
| Normalized Difference Vegetation Index(NDVI) | -32768.0 | 9938.0 | 188.9 | 6370.3 | https://www.ncei.noaa.gov/products/climate-data-records/normalized-difference-vegetation-index |

***Abbreviation:*** STD, standard deviation

(2) **For the concern of variables related to bat habitats, we indeed have considered them in the research because bats serve as an important host for the Ebola virus and their habitats can well demonstrate the changes of Ebola transmission. We devoted a lot of time to obtaining such data, including the Geographic Distribution of Ebola Virus Disease Outbreaks in Humans and Animals, as shown in the figure below, produced by the WHO Map Production: Health Statistics and Information Systems (HSI). However, it is really difficult to acquire the exact bat habitat data with specific locations geographically distributed across the whole Africa. Due to limitations in data acquisition, we did not use bat related variables in the current study. We have pointed out this problem in the discussion section. Hopefully in further study we can access such data to make more comprehensive analysis (Page 28, lines 425-428).**

8. **Section 2.2.3: Please provide citations and links for GPW, HII, DMSP and VIIRS data, as well as details of the variables extracted and their resolution. How was data cleaning done? What software did you use? What variables had to be cleaned? How did you establish the geospatial database?**
(1) **Thank you for bringing up this important question. The citations and links for GPW, HII, DMSP, and VIIRS data has been provided in Table 1 (Page 19) In addition, we have provided an official link (**https://www.worldclim.org/data/bioclim.html**) in the text that explains details of the variables extracted and their revolution (Page 18, lines 253).**
(2) **Ebola case data and multi-source raster data had to be cleaned (Page 13, lines 157-162).**

**For the case data, we firstly removed the irrelevant fields, then used Python to calculate the number of cases per province, and finally visualized the distribution of case points with ArcGIS, excluding data outside normal geographic bounds. In addition, to better match the distribution of Ebola case points with reality, we randomly generated additional points within a rectangular frame of 0.3 arc minutes around the center of each province. The number of those generated points depends on the proportion of cases in that area relative to the total number of cases in the epidemic.**

**For multi-source raster data, given the significant differences in their resolution and other aspects caused by sensors, we conducted normalization process using Python. We performed resampling, mask extraction of values, and cropping to ensure all rasters have a consistent resolution, row and column number, and pixel size. We then used ArcGIS software to standardize all of their geographic projections to the WGS84 coordinate system.**

(3) **After completing the data cleaning steps, we created a personal geospatial database in ArcGIS, and then set the coordinate system, resolution, tolerances. Next the processed data was imported to form three types of feature datasets (case point feature dataset, multi-source raster dataset, and national boundary vector dataset). Topological rules were added to the case point feature dataset and national boundary vector dataset, and quality checks were performed to complete the creation of the geospatial database. The content mentioned above is also supplemented in the text (Pages 13-14, lines 162-168).**
9. **Section 2.2.4: Please provide references for the methods and tools used? Did you use pre-existing software packages or write your own? What language did you use? What the correlation threshold used to remove variables? How sensitive are the results to the threshold used? The 13 variables listed here that are likely to influence Ebola transmission really should be part of the results, not methods. But it would be helpful to understand the full list of variables and what they mean. For example, how are temperature seasonality and isothermality defined? How is the coldest quarter defined, given that Sub-Saharan Africa cannot be divided into four distinct seasons?**
(1) **This is a very good comment. Regarding the references, we have provided one citation [24-27] for each method of variable filtering as follows (Page 14, lines 170-177):**
a. **The JackKnife method based on the Maxent model was cited from** *Guo Y, Zhang S, Tang S, Pan J, Ren L, Tian X, et al. Analysis of the prediction of the suitable distribution of Polygonatum kingianum under different climatic conditions based on the MaxEnt model. Frontiers in Earth Science. 2023;11.*
b. **The XGboost algorithm was cited from** *Snider B, McBean EA, Yawney J, Gadsden SA, Patel B. Identification of Variable Importance for Predictions of Mortality From COVID-19 Using AI Models for Ontario, Canada. 2021;9.*
c. **Spearman’s correlation coefficient was cited from** *Hauke J, Kossowski T. Comparison of Values of Pearson's and Spearman's Correlation Coefficients on the Same Sets of Data. Quaestiones Geographicae. 2011; 30: 87-93.* **and** *Kumar S, Stohlgren TJ, Chong GW. Spatial heterogeneity influences native and nonnative plant species richness. Ecology. 2006;87(12):3186-99.*
(2) **Regarding the methods, the JackKnife method was implemented based on the Maxent model using Maxent software V3.4.3, and additional details about this software has been included in the paper. The XGboost algorithm and Spearman’s correlation coefficient were conducted via Python programming as follows (Page 14, lines 170-171, 173-176):**

(3) **Regarding the threshold values, we set the correlation threshold between 0.75 and 0.85, considering variables with correlations above this threshold as highly correlated, and we selected only one from each group. Then we applied JackKnife and XGboost methods to respectively select 10 variables based on the order of training gain and contribution. After deleted the reduplicative variables, finally we obtained 13 variables as the input for the analysis. The final selection is less sensitive to the correlation threshold.**
(4) **Regarding paper structure, we really appreciate the reviewer’s suggestions that are very helpful to improve the overall organization and clarity of our manuscript. We have moved the content concerning variable selection from the *Method* section to the *Results* section, and the meanings of those variables can be found on the WorldClim official website, for which we have provided a link (**https://www.worldclim.org/data/bioclim.html**) in the text (Page 18, lines 244-253). Concerning the definition of 'the coldest quarter', especially in regions like Sub-Saharan Africa where the year cannot be distinctly divided into four seasons. According to the official documentation, ‘A quarter is a period of three months (1/4 of the year)’, it is reasonably infer that 'the coldest quarter' refers to the three consecutive months with the lowest temperatures in a year.**
10. **Section 2.3: Details on the model are way too sparse. I understand there might not be space to provide a huge amount of detail in the main text, but I think a brief description of the models, their differences and why one would want to combine them rather than perform a model selection is necessary. The details in the appendix are also much too sparse. A general outline of the models is provided, but is too generic. How do you use the models with your data specifically?**

**Thanks for your insightful and constructive comments. We have carefully considered those helpful suggestions and refined our descriptions of the models, particularly by supplement their strengths and weaknesses. To provide with a clear and succinct explanation, we created a Table (Table S1 in Appendix A) to demonstrate the characteristics and differences for each model. In addition, we have added how those models complement each other, as shown below, in the main text to further highlight the advantages of our proposed integrated model (Pages 14-15, lines 183-197). Also, in the appendix we have elaborated on each model's description, incorporating a deeper discussion tailored to our research context and data (Appendix B).**

*Table S1 in Appendix A shows the advantages and disadvantages of those four models and they complement each other in characteristics. Specifically, the Bioclim model can provide essential boundary conditions based on climatic extremes [28], serving as a foundational layer to define species' ecological limits. This can be offset by the Maxent model [29], which incorporates the complex, nonlinear ecological interactions that the Bioclim model may overlook, thus enriching the predictive model with greater ecological depth. On the other hand, simplistic the Domain model [30], can offer valuable preliminary insights especially in data-limited situations, establishing a baseline distribution hypothesis that can be refined. Finally, the iterative optimization processes of the GARP model can better refine the initial predictions by systematically improving accuracy through complex rule handling [31]. Therefore, the integrated model by combining these approaches can not only consolidate basic climatic thresholds and ecological interactions but also increase the precision and adaptability in predicting specie distribution under varying environmental conditions.*

**Table S1.** The advantages and disadvantages of Maxent, Bioclim, Domain, and GARP models

| **Model** | **Advantages** | **Disadvantages** |
| --- | --- | --- |
| Maxent [31] | - Efficiently handles sparse data and complex interactions - Suitable for high-dimensional and nonlinear data - Strong regularization prevents overfitting | - Numerous parameters, complex model adjustment - Requires substantial data for optimal performance |
| Bioclim [30] | - Simple operation, easy to understand and implement - Stable predictions based on extreme climate values | - Ignores non-climatic ecological factors - Unable to identify interactions among climatic variables |
| Domain [32] | - Intuitively reflects species-environment similarity - Suitable for data-limited rare species | - Sensitive to outlier data, easily disturbed - Simplified models may overlook important environmental variables |
| GARP [33] | - Iterative optimization enhances model performance - Effectively handles complex non-linear rules | - High computational and resource demands - Results may vary significantly |

11. **How is the model trained? What formula do you use to calculate the weights? Thanks very much for your concern. How the models were trained are as follows (Appendix B):**
(1) **For the Maxent model, we employed Maxent software V3.4.3, a Java-based tool designed by Steven J. Phillips from Columbia University for predicting the potential distribution of species. We utilized the R package ENMeval for tuning parameters. ENMeval allows running ecological niche models across all combinations of user-defined settings and performs cross-validation to evaluate the models. We trained the model based on the optimal parameters obtained.**
(2) **For the Bioclim and Domain models, we based on DIVA-GIS software, firstly imported the case point data and the prediction range in vector format, then used the DIVA-GIS functionality to merge the environmental raster data into one stack file, based on which we trained and analyzed the models.**
(3) **For the GARP model, we used DesktopGARP 1.1.3 software. Initially, we converted tif raster files from the database into asc files, created a mask file based on the scope, rows, columns, and pixel size of the raster files, then converted the asc files into raw files. Subsequently, we input those files along with the case point vector data into the software for model training. We have appropriately supplemented the methods of model construction and training in the appendix.**

**Regarding the model weights, the reviewer’s concern is indeed very valuable. How to determine the weights in the model integration process needs to not only consider accuracy but also the distinct characteristic of each model. Therefore, it is challenging to use quantitative methods. Hence, we applied the Analytic Hierarchy Process (AHP) to determine the weight by taking into account a group of factors including model accuracy, nonlinear data handling capabilities, complexity, data volume requirements, and generalizability. We obtained the relative importance of those factors in conjunction with our specific research data, and then calculated the final weights.** **We have supplemented such description in the manuscript (Page 16, lines 220-222) and provided a reference of relevant literature** (*Ge Z, Liu Y. Analytic Hierarchy Process Based Fuzzy Decision Fusion System for Model Prioritization and Process Monitoring Application. IEEE Transactions on Industrial Informatics. 2019;15(1):357-65.*) **as follows:**

*The weights* $W_{i}$ *were derived through the Analytic Hierarchy Process (AHP), comprehensively considering factors such as model accuracy, nonlinear data handling capabilities, complexity, data volume requirements, and generalizability [32].*

**<Results>**
**Reviewer #1: (No Response)
Reviewer #2:**

12. **It is very hard to assess the results given that the figures provided do not generally correspond with the figures referred to in the text.**

**Thank you for pointing out this problem. This is our mistake, and we sincerely apologize for the error in numbering figures. We have carefully examined the numbers of figures in the text and corrected the mistakes to ensure all the numbers correspond with the right figure.**

13. **Section 3.1: The first paragraph on reclassification should really be part of the methods section, and more details on how this reclassification is done are needed. Figure 2 does not have subfigures labelled a, b and c. Figures 3a, b and c are never referred to - maybe rethink whether they need to be included? How were the weights chosen calculated? Please label your figures carefully - I'm guessing figures s2 and s3 should actually be figures 5 and 6? Are the countries in red bars actually at risk of Ebola outbreaks? Have you taken into account bat habitats and whether bats live there?**
(1) **Thank you for your professional comment. According to the reviewer’s suggestion, we have moved the first paragraph on reclassification to the methods section, and also we have added more details on how this reclassification is done, which can be found on Page 16, lines 214-218 as follows:** *Since the Maxnet, Bioclim, and Domain models all derived specific values while the GARP model provides logistic variables, it is necessary to standardize these different results for effective comparison. We reclassified the outputs from the first three models into five uniform intervals from 0 to 1, each interval at 0.2, to align with the logistic variables from the GARP model.*
(2) **We sincerely apologize again for the confusion of figure number, we have carefully examined the figure number and corrected the mistakes to ensure all number correspond with the right figure referred to in the text. In the original version, we mistakenly labeled Figure 3 as Figure 2. This led to references in the text to subfigures a, b, and c for Figure 2 that did not exist, while the subfigures a, b, and c for Figure 3 were not mentioned in the text. We have corrected these errors to ensure clarity and accuracy in the revised version.**
(3) **Regarding the weights, we primarily used the Analytical Hierarchy Process (AHP), which has been explained in our aforementioned response to Methods (Comment 11). We have added such supplementary description in the manuscript and provided a citation (Page 16, lines 220-222).**
(4) **Concerning the red bars, by integrating the predictive results with population density grids, we estimated the total population affected in each country within the high-risk areas. Based on our experimental findings, the red bars demonstrate the countries that have not reported Ebola cases previously but are still at potential risk. Those areas need paying special attention and taking appropriate health measures to prevent Ebola epidemic.**
(5) **For the constructive and valuable concern of bat habitats, we have explained it in our aforementioned response to Methods (Comment 7). Bat habitats can well demonstrate the changes of Ebola transmission. We have devoted a lot of time to obtaining such data, however, it is really difficult to acquire the exact bat habitat data with specific locations geographically distributed across the whole Africa. Due to limitations in data acquisition, we did not use bat related variables in the current study. We have pointed out this problem in the discussion section. Hopefully in further study we can access such data to make more comprehensive analysis (Page 28, lines 425-428).**
14. **Section 3.2: How was the impact factor analysis done? More detail on how the variable interact with risk is needed. The Jackknife results are in figure 4, not figure 3.**
(1) **Thanks for your insightful question. The Contribution and Permutation Importance of variables, as well as the Jackknife results in Section 3.2, were all generated using Maxent software version 3.4.3. For the Jackknife method, two approaches were used to analyze the impact factor. If the model is built considering only a single variable, the blue bar in Figure 4 represents the interaction between that variable and the risk. Conversely, if a variable is excluded from the model, the green bar in Figure 4 reflects the interaction when that variable is excluded. We have added this explanation in the manuscript (Page 22, lines 308-313) as follows:**

*Jackknife testing (Figure 6) based on Maxent software 3.4.3, applied two methods to evaluate the impact of environmental variables on Ebola virus models. The first method involves using only one variable at a time, highlighting the direct impact of variables like bio3, bio4, bio11, bio12, GPP, and GPW on model performance metrics. The second one excludes one variable to assess its unique contribution, revealing that the exclusion of GPW, LCCS, and bio12 significantly reduced model accuracy.*

(2) **The Jackknife results should correspond to Figure 4, and we have corrected it.**
15. **Reference 20 is probably not correct.**

**Thanks very much for your insightful comments. We sincerely apologize for the confusion caused by this citation. Our original intention was to refer the study by Guo et al. (2023) to support why and how we applied Jackknife method in our analysis, as well as the principle and suitability. We hoped this reference could help verify our interpretation of the Jackknife results. This citation is more of method perspective, and less related to Ebola itself. We have realized that this citation put here may be inappropriate and could cause confusion. Therefore, we have removed it from the reference list (Page 22, lines 308-313).**

16. **Section 3.3: I think figure S4 should be figure S1 and figure 4 should be figure 7? Please describe the relationship between the variables and outbreak risk? Are they significant? It is interesting that risk is high for low and high LCCS but not intermediate values - do you have any insight into why? Also, please replace bio3, bio4, bio6, bio7, bio11, bio12, GPP, GPW, HII, LCCS, light and NDVI with more descriptive names. What do the range of these variables represent? What are their units?**
(1) **Thanks for your very kind revision. We sincerely apologize again for the confusion caused by the incorrect figure numbering. We have thoroughly reviewed and corrected all figure numbers in the manuscript to ensure accuracy.**
(2) **The relationships between the variables and outbreak risk are significant, and their description have been added to Page 24, lines 338-350 as follows:**
a. *The ecological adaptability of the Ebola virus is inversely related to temperature seasonality (bio4) but positively correlates with higher winter average temperatures and annual precipitation (bio6, bio12).*
b. *Greater population densities enhance the ecological adaptability of the Ebola virus (GPW). However, this adaptability exhibits a bell-shaped curve in relation to economic development, increasing initially before diminishing in more economically advanced regions (HII).*
c. *The ecological adaptability of the Ebola virus shows an initial increase followed by a decline in response to rising vegetation levels, peaking in areas with moderate vegetation coverage.*
d. *The Ebola virus adaptability responds positively to various land covers, with specific codes representing rainfed cropland (10), mosaic cropland (30), broadleaf deciduous forests (60), flooded forests (170), and urban zones (190) (LCCS).*
(3) **The relationship between LCCS and disease risk was mentioned in the Discussion section (Page 27 lines 414-421). LCCS is a discrete variable where the numbers serve as identifiers for different land use types. These numbers do not have inherent meaning themselves; they simply correspond to specific categories of land use. Therefore, we cannot precisely determine the reasons why low and high LCCS values lead to high risk. We referenced Redding et al. (2019)'s work, which suggests an indirect link between land use and host distribution. Land use types that potentially contain hosts and areas of human-host contact, such as rainfed cropland, mosaic cropland, broadleaf deciduous forests, and flooded forests, are associated with a higher probability of zoonotic transmission and consequently, increased disease risk. Additionally, urban zones, with their frequent human interactions, exhibit a higher likelihood of human-to-human transmission.**
(4) **According to the scientific writing convention, we have provided the descriptive full names for all the variables when they firstly appear in the manuscript (Pages 17-18, lines 244-253), and then we used abbreviations of those aforementioned variables in the subsequent text. Detailed information regarding the meaning, range, and units of the variables has been provided in Table 1 (Page 19).**

**<Conclusions>**
**Reviewer #1: (No Response)
Reviewer #2:**

17. **The papers by Pigott and Redding should really be discussed in the introduction.**

**Thanks, according to the reviewer’s suggestion, we have added the discussion of the paper by Pigott and Redding in the introduction (Page 9-10, Citation 14 and 16).**

18. **Patterns 1-4 should really be described in the results, not in the discussion. Their significance and relevance can be assessed in the discussion.**

**This is a very good comment. According to the reviewer’s suggestion, we have moved the description of Patterns 1-4 to the results section (Page 24, lines 338-350), while keeping the assessment of their significance and relevance in the discussion (Page 26-27, lines 391-421).**

**<Summary and General Comments>
Reviewer #1:**

19. **It is advisable to simplify the description of EVD in the first paragraph (Line 59-63) to make it more succinct.**

**Thank you for your valuable comments. We have revised the description in the first paragraph accordingly to make it more succinct (Page 9, lines 57-60).**

20. **The literature review of different models in the application of EVD research needs to be shortened and succinct (Line 70-86).**

**Thanks, we really appreciate your suggestion and have revised the literature review on the application of EVD research models to make it shorter and more succinct (Pages 9-10, lines 67-81).**

21. **Some of the figures need to be improved by using high resolution. Especially when it comes to zooming in those figures, a part of information looks really blur.**

**Thanks for your comment. The blurring of our images was due to the automatic compression when converting them to PDF. We have now switched the image format from PNG to TIFF, which has significantly improved the quality of the images in the PDF. The improved images get clearer even when zoomed in.**

22. **References need to be given to the four ecological niche models in Line 180.**

**Thanks for your professional comment. We have added descriptions for the four niche models, and provided references for each model (Pages 14-15, lines 185-197) as follows:**

(1) **The Maxent model was cited from** *Phillips SJ, Anderson RP, Schapire RE. Maximum entropy modeling of species geographic distributions. Ecological Modelling. 2006;190(3):231-59.*
(2) **The Bioclim model was cited from** *Booth TH, Nix H, Busby JR, Hutchinson MFJD, Distributions. bioclim: the first species distribution modelling package, its early applications and relevance to most current MaxEnt studies. 2014;20.*
(3) **The Domain model was cited from** *Carpenter G, Gillison AN, Winter J. DOMAIN: a flexible modelling procedure for mapping potential distributions of plants and animals. Biodiversity & Conservation. 1993;2(6):667-80.*
(4) **The GARP model was cited from** *Peterson A, Papes M, Eaton M. Transferability and model evaluation in ecological niche modeling: A comparison of GARP and Maxent. Ecography. 2007; 30:550-60.*
23. **Since the full names of different variables have been mentioned in Line 169-174, there is no need to repeat the full name for some variables in Line 279-281.**

**Thanks for your comment. According to the reviewer’s suggestion and the scientific writing convention, we have provided the descriptive full names for all the variables when they firstly appear in the manuscript (Pages 17-18, lines 244-253), and then we used abbreviations of those aforementioned variables in the subsequent text.**

24. **It is suitable to add a Data availability statement at the end of the research part to provide support for researchers who have an interest on this study.**

**Thanks for your comment. We have added a Data Availability Statement at the end of the research section to support researchers interested in this study. (Page 32, lines 513-516)**

25. **One or more pertinent/related scholarly references could be cited in the current reference list.**

**Thanks, according to the reviewer’s suggestion, we have added more references to our paper as follows:**

(1) *Judson SD, Fischer R, Judson A, Munster VJ. Ecological Contexts of Index Cases and Spillover Events of Different Ebolaviruses. PLOS Pathogens. 2016;12(8):e1005780.*
(2) *Buceta J, Johnson K. Modeling the Ebola zoonotic dynamics: Interplay between enviroclimatic factors and bat ecology. PLOS ONE. 2017;12(6):e0179559.*
(3) *Lee-Cruz L, Lenormand M, Cappelle J, Caron A, De Nys H, Peeters M, et al. Mapping of Ebola virus spillover: Suitability and seasonal variability at the landscape scale. PLOS Neglected Tropical Diseases. 2021;15(8):e0009683.*
(4) *WHO (World Health Organization) [Internet]. Ebola outbreak in Democratic Republic of the Congo declared over; c2024[cited 2024 Aug 8]. Office of International Medicine. Available from: https://www.who.int/southeastasia/publications/i/item/9789290211495..*
(5) *Weyer J, Grobbelaar A, Blumberg L. Ebola virus disease: history, epidemiology and outbreaks. Current infectious disease reports. 2015;17(5):480.*
(6) *Guo Y, Zhang S, Tang S, Pan J, Ren L, Tian X, et al. Analysis of the prediction of the suitable distribution of Polygonatum kingianum under different climatic conditions based on the MaxEnt model. Frontiers in Earth Science. 2023;11.*
(7) *Snider B, McBean EA, Yawney J, Gadsden SA, Patel B. Identification of Variable Importance for Predictions of Mortality From COVID-19 Using AI Models for Ontario, Canada. 2021;9.*
(8) *Hauke J, Kossowski T. Comparison of Values of Pearson's and Spearman's Correlation Coefficients on the Same Sets of Data. Quaestiones Geographicae. 2011;30:87-93.*
(9) *Ge Z, Liu Y. Analytic Hierarchy Process Based Fuzzy Decision Fusion System for Model Prioritization and Process Monitoring Application. IEEE Transactions on Industrial Informatics. 2019;15(1):357-65.*
26. **Please double check the grammar mistake and carefully revise the English writing.
    Thanks, we really appreciate your valuable comments and have double checked the grammar mistake and carefully revised the English writing throughout the manuscript. In addition, we have also rewritten some of the sentences for better understanding.**

**Reviewer #2:**

27. **The introduction fails to motivate the study and demonstrate its novelty. The authors discuss the current state of Ebola modelling by solely discussing transmission modelling (and citing a very limited subset of methods and papers at that) - however, they do not really mention previous ecological models in this section. Based on this, the reader may think that this is the first time an ecological niche modelling (ENM) approach has been used in the context of Ebola outbreaks. In the discussion section, the authors do refer to two other ecological models which agree with their findings. However, these models really need to be discussed in the introduction section. What has been done before? What approaches have been used? What were these studies' limitations and how does your model specifically advance our understanding of the Ebola virus' ecological niche? In addition to Redding et al and Piggott et al, other papers to consider include: Judson et al (PLOS Pathogens 2016), Lee-Cruz et al (PLOS NTDs 2021), Buceta et al (PLOS One 2017), to name just a few.
    Thanks for your very constructive comments and providing with relevant academic papers to improve our research, which we really appreciate. According to the reviewer’s suggestion, we have carefully read the recommended literature and revised our introduction by comparing to those cited papers and added a discussion of those model. Also we talked the need to develop an integrated model for Ebola virus niche research. For a detailed comparison of each model and the rationale behind the integrated model, please refer to the Methods section (Pages 14-15, lines 183-197), where we elaborate it more specifically. The revised content of the introduction section is as follows (Pages 9-10, lines 67-97):**

*Previous scholars have been dedicated to researching virus spread through interactions between the susceptible agents and infected animals or humans. A variety of models have been developed or improved by continuously optimizing parameters [9]. For example, ecological niche models, incorporating environmental factors as covariates and using presence-only data, can be used to identify diverse habitats suitable for the spillover of different ebolaviruses [10]. From a zoonotic perspective, the Susceptible-Infectious-Recovered (SIR) compartmental model has also been deployed to explore the dynamics of Ebola transmission between species and simulate Ebola trends [11-14]. Also, the Geographic Information System-based multicriteria evaluation (GIS-MCE) model can generates maps of risk area for Ebola spillover by taking account the spatial and temporal variability [15]. In addition, epidemiological compartmental models are used to forecast the spatial and temporal risks associated with Ebola Virus Disease [16]. And time-series regression models have significant potential to extract the filovirus distribution for improving the overall accuracy of traditional models [17-19].*

*However, those aforementioned traditional mathematical models can hardly incorporate the spatial variation of Ebola pathogens to accurately reveal its potential outbreak source and sink areas. Especially relying on a single specific model may cause a decrease of the robustness and accuracy of the results due to the inherent limitations. Besides, the conventional models have paid little attention to simultaneously examining the impacts of both natural and anthropogenic factors on the host movement from a spatiotemporal perspective. Moreover, most relevant studies fail to use a comprehensive spatiotemporal dataset on potential influencing factors and the latest case data, such as from the 2022 Uganda outbreak based on a unified spatiotemporal scale.*

*Therefore, this study is purposed to propose a synthesized spatiotemporal framework to quantitatively explore the dynamic distribution of Ebola outbreaks in Africa, as well as the driving impact from different factors. A comprehensive and robust analysis can be provided by strategically integrating four distinct ecological models and incorporating an extensive dataset of multi-facet environmental factors and the most current case information.*

28. **More care needs to be taken with citations. There are some statements made in the paper that really need to be backed up with citations. Other papers are miscited. For example, a paper by Merler at el is cited to show that vegetation and climate both impact Ebola transmission; however this paper is an agent-based model on Ebola transmission that assesses the impact of non-pharmaceutical interventions and makes no mention of climate. Similarly, Guo et al is cited to suggest that specific variables are important for Ebola transmission, but is really about the spatial distribution of a plant species.**

**Thank you for your valuable comment and pointing out the issues with the citations in this manuscript. Those are our mistakes and we are really sorry for this. We have thoroughly examined the references and made corrections to ensure that all statements are accurately supported by the appropriate citations. For the citation of Merler et al. (2015), we identified that the reference number was incorrect, and it has now been corrected. Regarding the citation of Guo et al. (2023), we have decided to remove it to prevent any potential misunderstanding, which has been addressed in previous response to Comment 4. We appreciate your guidance and support, which have helped a lot to improve the accuracy and clarity of our manuscript.**

29. **Much more care needs to be taken with figures. The submitted article contained two lists of figure legends: one for figures 1-4 and S1-S3, and one for figures 1-7 and S1. However, none of these seem to match up with the figures accompanying the paper (labelled 1 to 7 and S1). For example, the first list of figure legends says that figure 3 shows the results of a Jackknife test, while the second set of figure legends says that figure 6 shows the Jackknife test. Having looked at the figures, it is actually figure 4 that shows the results of the Jackknife test. It is virtually impossible for the reader to understand whether the results support the conclusions when some figures are never referred to, some figures that are referred to are not shown, and figures are generally mislabelled.**

**Thanks again for pointing out this problem. This is our mistake, and we sincerely apologize for the error in numbering figures. We have carefully examined all figure number and corrected the mistakes to ensure all numbers correspond with the right figure throughout the paper. All figures have been referred to and all labels have been corrected.**

30. **More care also needs to be taken with the paper organization: the methods section contains some results, the results section contains some methods, and results are also presented in the discussion.
    Thank you for your comment regarding the organization of our paper. According to the reviewer’s suggestion, we have made all the corresponding changes to reorganize different sections. The discussion of selected variables has been moved to the Results section (Pages 17-18, lines 244-253), and the reclassification description has been put into the Methods section (Page 16, lines 214-218). In addition, we have shifted the discussion of Pigott and Redding's papers to the Introduction (Pages 9-10, lines 67-81), and placed the analysis of trends in response curves in the Results section (Page 24, lines 338-350). Hopefully those changes can ensure a clearer and more rigorous structure throughout the manuscript. s**
31. **I would be amenable to reading a revised version of this paper as I believe it is an important topic. However, it is not ready for publication in its current form.
    Finally, we really appreciate your comments and support which are so valuable to our manuscript. We have really improved our academic writing through this whole revision process. Hopefully this revised version can address all your concerns of the research. Thanks very much again!**

14-Aug-2024

Manuscript ID: PNTD-D-24-00256R1

**Reply to Editors**

**Editor’s comments:**

Dear Dr Li,
Thank you for submitting your paper, "Assessing the ecological resilience of Ebola virus in Africa and potential influencing factors based on a synthesized model", to PLOS Neglected Tropical Diseases. We have checked over your submission and, before we can assign your manuscript to the Editor, we ask that you first please make the following modifications:

1) Please include your title page at the beginning of your manuscript file that lists full author names and institute addresses. This should not be uploaded as a separate file.

**Thanks and done. We have included title page at the beginning of the manuscript file that lists full author names and institute addresses as requested (Pages 1-5).**

2) Please upload all main figures as separate Figure files in .tif or .eps format. For more information about how to convert and format your figure files please see our guidelines: <https://journals.plos.org/plosntds/s/figures>

**Thanks and done. We have re-uploaded all main figures as separate Figure files in .tif format.**

3) We have noticed that you have uploaded Supporting Information files, but you have not included a list of legends. Please add a full list of legends for your Supporting Information files after the references list.

**Thanks and done. We have added a full list of legends for the Supporting Information files after the references list as follows (Page 35, lines 618-628):**

*Appendix A：Supplemental table and figures.*

- *Figure S1. Multiple data sources, including Ebola epidemic case data, surveyed attribute data, remote sensing data, and spatial vector boundaries, and establishment of a geodatabase. The base layers of the map were obtained from the openly available source via the Ministry of Natural Resources Standard Land Service of China (http://bzdt.ch.mnr.gov.cn/index.html).*
- *Figure S2. Heat map of spearman coefficients for each variable illustrating the correlations among variables.*
- *Table S1. The advantages and disadvantages of Maxent, Bioclim, Domain, and GARP models*

*Appendix B: Characteristics and introduction of the models used in this study.*

4) Some material included in your submission may be copyrighted. According to PLOSu2019s copyright policy, authors who use figures or other material (e.g., graphics, clipart, maps) from another author or copyright holder must demonstrate or obtain permission to publish this material under the Creative Commons Attribution 4.0 International (CC BY 4.0) License used by PLOS journals. Please closely review the details of PLOSu2019s copyright requirements here: PLOS Licenses and Copyright. If you need to request permissions from a copyright holder, you may use PLOS's Copyright Content Permission form.
Please respond directly to this email and provide any known details concerning your material's license terms and permissions required for reuse, even if you have not yet obtained copyright permissions or are unsure of your material's copyright compatibility. Once you have responded and addressed all other outstanding technical requirements, you may resubmit your manuscript within Editorial Manager. 
Potential Copyright Issues:

i) S1 Figure. Please confirm whether you drew the images / clip-art within the figure panels by hand. If you did not draw the images, please provide (a) a link to the source of the images or icons and their license / terms of use; or (b) written permission from the copyright holder to publish the images or icons under our CC BY 4.0 license. Alternatively, you may replace the images with open source alternatives. See these open source resources you may use to replace images / clip-art:
- <https://commons.wikimedia.org/>

- <https://openclipart.org/>

If you did not draw the images, please provide (a) a link to the source of the images or icons and their license / terms of use;

**Thank you very much for your thorough review of the manuscript. We drew the S1 figure using ArcGIS software (version 10.8), using icons with open source downloaded from the *iconfont* website:** [**https://www.iconfont.cn/**](https://www.iconfont.cn/help/index?spm=a313x.help_detail.i3.25.30f63a81fe4tTa)**. The user service agreement could be seen at:** [**https://terms.alicdn.com/legal-agreement/terms/platform_service/20220704165734807/20220704165734807.html?spm=a313x.collections_detail.i7.1.1e2d3a81TLH1kc**](https://terms.alicdn.com/legal-agreement/terms/platform_service/20220704165734807/20220704165734807.html?spm=a313x.collections_detail.i7.1.1e2d3a81TLH1kc)**.**

ii) Figure 1 to Figure 5 and S1 Figure. Please (a) provide a direct link to the base layer of the map (i.e., the country or region border shape) and ensure this is also included in the figure legend; and (b) provide a link to the terms of use / license information for the base layer image or shapefile. We cannot publish proprietary or copyrighted maps (e.g. Google Maps, Mapquest) and the terms of use for your map base layer must be compatible with our CC BY 4.0 license.
Note: if you created the map in a software program like R or ArcGIS, please locate and indicate the source of the basemap shapefile onto which data has been plotted.
If your map was obtained from a copyrighted source please amend the figure so that the base map used is from an openly available source. Alternatively, please provide explicit written permission from the copyright holder granting you the right to publish the material under our CC BY 4.0 license.
If you are unsure whether you can use a map or not, please do reach out and we will be able to help you. The following websites are good examples of where you can source open access or public domain maps:
* U.S. Geological Survey (USGS) - All maps are in the public domain. (<http://www.usgs.gov>)
* PlaniGlobe - All maps are published under a Creative Commons license so please cite u201cPlaniGlobe, <http://www.planiglobe.com>, CC BY 2.0u201d in the image credit after the caption. (<http://www.planiglobe.com/?lang=enl)>
* Natural Earth - All maps are public domain. (<http://www.naturalearthdata.com/about/terms-of-use>).

**Thanks for your insightful and constructive comments. We have added a direct link to the base layer of the map in the figure legends for Figure 1 to Figure 5 and S1 Figure. The map data used in the study were obtained from the openly available source via the Ministry of Natural Resources Standard Land Service of China(**[**http://bzdt.ch.mnr.gov.cn/index.html**](http://bzdt.ch.mnr.gov.cn/index.html)**). The basemap shapefile was drawn with reference to the world standard map (No. GS(20)1666). The map contains layers such as continents, country or region borders, cities, administrative divisions by country, lakes, rivers, latitudes and longitude. In this research, the projection coordinate system of map data is WGS_1984_Plate_Carree, and the geographic coordinate system is GCS_WGS_1984.**

5) In the online submission form, you indicated that The original research data for this paper can be obtained by sending a request email to the corresponding author([liukun5959@qq.com](mailto:liukun5959@qq.com)). All PLOS journals now require all data underlying the findings described in their manuscript to be freely available to other researchers, either
1. In a public repository
2. Within the manuscript itself
3. Uploaded as supplementary information.
This policy applies to all data except where public deposition would breach compliance with the protocol approved by your research ethics board. If your data cannot be made publicly available for ethical or legal reasons (e.g., public availability would compromise patient privacy), please explain your reasons by return email and your exemption request will be escalated to the editor for approval. Your exemption request will be handled independently and will not hold up the peer review process, but will need to be resolved should your manuscript be accepted for publication. One of the Editorial team will then be in touch if there are any issues.
**Thanks for your insightful and constructive comments. All data from this study are publicly available and are described below (Page 32, lines 513-516):**

*Data Availability:*

*The authors confirm that all data underlying the findings are fully available without restriction. All relevant data are within the paper and its Supporting Information files.*

6) Please amend your detailed Financial Disclosure statement. This is published with the article. It must therefore be completed in full sentences and contain the exact wording you wish to be published.

1) State what role the funders took in the study. If the funders had no role in your study, please state: "The funders had no role in study design, data collection and analysis, decision to publish, or preparation of the manuscript.

2) If any authors received a salary from any of your funders, please state which authors and which funders.
If you did not receive any funding for this study, please simply state: The authors received no specific funding for this work.
**Thanks. This work was partly supported by grants from the National Natural Science Foundation of China [grant number 42201448 to Li Shen and 82273689 to Zhongjun Shao], and Natural Science Foundation of Hubei Province [grant number 2022CFB610 to Li Shen].**

7) Please ensure that the funders and grant numbers match between the Financial Disclosure field and the Funding Information tab in your submission form. Note that the funders must be provided in the same order in both places as well. Currently, order of grants are different in both places.
**Thanks. We have rechecked and corrected the funding information as show in point (6).**

16-Aug-2024

Manuscript ID: PNTD-D-24-00256R1

1) We note that your Data Availability Statement is currently as follows: [The authors confirm that all data underlying the findings are fully available without restriction. All relevant data are within the paper and its Supporting Information files.]
   Please confirm at this time whether or not your submission contains all raw data required to replicate the results of your study. Authors must share the “minimal data set” for their submission. PLOS defines the minimal data set to consist of the data required to replicate all study findings reported in the article, as well as related metadata and methods (https://journals.plos.org/plosone/s/data-availability#loc-minimal-data-set-definition).
   For example, authors should submit the following data:
   - The values behind the means, standard deviations and other measures reported;
   - The values used to build graphs;
   - The points extracted from images for analysis.
   Authors do not need to submit their entire data set if only a portion of the data was used in the reported study.
   If your submission does not contain these data, please either upload them as Supporting Information files or deposit them to a stable, public repository and provide us with the relevant URLs, DOIs, or accession numbers. For a list of recommended repositories, please see https://journals.plos.org/plosone/s/recommended-repositories.
   If there are ethical or legal restrictions on sharing a de-identified data set, please explain them in detail (e.g., data contain potentially sensitive information, data are owned by a third-party organization, etc.) and who has imposed them (e.g., an ethics committee). Please also provide contact information for a data access committee, ethics committee, or other institutional body to which data requests may be sent. If data are owned by a third party, please indicate how others may request data access.

**We’re sorry for ignoring the data sharing issue. Our submission contains all raw data required to replicate the results of the study. All data could be queried and downloaded through the website provided in the corresponding position of the text of the text. Detailed methods could also be found in the main text or supplemental material. We also shared the data cleaned and collated to build models, tables and figures to meet the criterion of “minimal data set” for submission through the *Researchgate* website at:** [**https://www.researchgate.net/publication/383181773_Assessing_the_ecological_resilience_of_Ebola_virus_in_Africa_and_potential_influencing_factors_based_on_a_synthesized_model/**](https://www.researchgate.net/publication/383181773_Assessing_the_ecological_resilience_of_Ebola_virus_in_Africa_and_potential_influencing_factors_based_on_a_synthesized_model/)**. If this link reaches a error page, please visit the homepage of the corresponding author Rui Li, which located at:** [**https://www.researchgate.net/profile/Li-Rui-73**](https://www.researchgate.net/profile/Li-Rui-73)**. We amended the detailed Data availability statement as follow (Page 32, lines 515-518):**

**The authors confirm that all data underlying the findings are fully available without restriction. All relevant data are within the paper and its Supporting Information files. Publicly available data (CC BY 4.0) has been uploaded to the *Researchgate* (**[**https://www.researchgate.net/profile/Li-Rui-73**](https://www.researchgate.net/profile/Li-Rui-73)**).**

2) Please amend your detailed Financial Disclosure statement. This is published with the article, therefore should be completed in full sentences and contain the exact wording you wish to be published.
   i). State what role the funders took in the study. If the funders had no role in your study, please state: “The funders had no role in study design, data collection and analysis, decision to publish, or preparation of the manuscript.”
   ii). If any authors received a salary from any of your funders, please state which authors and which funders.

**We’re sorry for this issue. We have completed the detailed Financial Disclosure statement in full sentences as follow:**

***• Funding: This work was partly supported by grants from the National Natural Science Foundation of China [grant number 42201448 and 82273689], and Natural Science Foundation of Hubei Province [grant number 2022CFB610]. The funders had no role in study design, data collection and analysis, decision to publish, or preparation of the manuscript.***

3) Some material included in your submission may be copyrighted. According to PLOS’s copyright policy, authors who use figures or other material (e.g., graphics, clipart, maps) from another author or copyright holder must demonstrate or obtain permission to publish this material under the Creative Commons Attribution 4.0 International (CC BY 4.0) License used by PLOS journals. Please closely review the details of PLOS’s copyright requirements here: PLOS Licenses and Copyright. If you need to request permissions from a copyright holder, you may use PLOS's Copyright Content Permission form. Please respond directly to this email and provide any known details concerning your material's license terms and permissions required for reuse, even if you have not yet obtained copyright permissions or are unsure of your material's copyright compatibility. Once you have responded and addressed all other outstanding technical requirements, you may resubmit your manuscript within Editorial Manager.
Potential Copyright Issues:

(1) [Figure 1 to 5, S1 Figure]: Thank you for stating" The map data used in the study were obtained from the openly available source via the Ministry of Natural Resources Standard Land Service of China(<http://bzdt.ch.mnr.gov.cn/index.html>). This link reaches a error page. Please amend this to a working link. The following websites are good examples of where you can source open access or public domain maps:
* U.S. Geological Survey (USGS) - All maps are in the public domain. (http://www.usgs.gov)
* PlaniGlobe - All maps are published under a Creative Commons license so please cite u201cPlaniGlobe, http://www.planiglobe.com, CC BY 2.0u201d in the image credit after the caption. (http://www.planiglobe.com/?lang=enl)
* Natural Earth - All maps are public domain. (<http://www.naturalearthdata.com/about/terms-of-use/>).

**We’re sorry for this issue. The Ministry of Natural Resources Standard Land Service of China (new link:** [**http://bzdt.ch.mnr.gov.cn/**](http://bzdt.ch.mnr.gov.cn/)**) maybe could not be accessed outside China. But we have checked the maps sources, they are the same as made with Natural Earth (**[**https://www.naturalearthdata.com/downloads/50m-cultural-vectors/**](https://www.naturalearthdata.com/downloads/50m-cultural-vectors/)**). We have re-drew and upload the mentioned figures, and this has no effect on the presentation of the results. Thus we amend the statement as follow: The map data used in the study were obtained from the openly available source via the Natural Earth (https://www.naturalearthdata.com/downloads/50m-cultural-vectors/).**

(2) [Figure S1]: Thank you for stating"We drew the S1 figure using ArcGIS software (version 10.8), using icons with open source downloaded from the iconfont website: https://www.iconfont.cn/. The user service agreement could be seen at: https://terms.alicdn.com/legal-agreement/terms/platform_service/20220704165734807/20220704165734807.html?spm=a313x.collections_detail.i7.1.1e2d3a81TLH1kc.
https://terms.alicdn.com/legal-agreement/terms/platform_service/20220704165734807/20220704165734807.html?spm=a313x.collections_detail.i7.1.1e2d3a81TLH1kc : This link reaches a error page. Please amend this to a working link.
Alternatively, you may replace the images with open source alternatives. See these open source resources you may use to replace images / clip-art:
- https://commons.wikimedia.org
- https://openclipart.org/"
**We’re sorry for this issue again. The website for user service agreement could not be accessed outside China, while the content is all written in Chinese. To avoid copyright issues, we removed icons downloaded from the *iconfont* website, and re-drew the Figure S1, the rest icons are all inside *Microsoft Office Word*.**
