# Supplementary material for: Assessing the ecological resilience of Ebola virus in Africa and potential influencing factors based on a synthesized model
Source: PLoS Negl Trop Dis. 2025 Feb 7;19(2):e0012843. doi: 10.1371/journal.pntd.0012843 (PMC11805440; doi:10.1371/journal.pntd.0012843)
Supplement: Attachment — Submitted filename: Response to Reviewers.docx [file pntd.0012843.s005.docx]

2-Dec-2024

Dear Editor,

Thank you so much for your kind consideration of our manuscript entitled ***‘Assessing the ecological resilience of Ebola virus in Africa and potential influencing factors based on a synthesized model****’* (Manuscript reference number: **PNTD-D-24-00256**) and for sharing the insightful review comments which have helped improving our manuscript a lot.

It has been about ten months since we submitted the first version of our manuscript, and this is our second-round major revision. We have carefully considered all the comments and suggestions from both the editor and reviewer, and then revised the manuscript accordingly. Enclosed please find our point-by-point responses to their comments and suggestions. All the corrections have been highlighted in yellow of the revised manuscript. We hope you will find our manuscript appropriated for publication in ***PLOS Neglected Tropical Diseases.***

As before, this manuscript is not under consideration for publication by any other journal or medium, and all authors have agreed to its publication. We really appreciate your support and help in our manuscript, and look forward to your reply.

Sincerely yours,

Kun Liu, PhD

Department of Epidemiology, School of Public Health,

Air Force Medical University,

169 Chang-Le Street, Xincheng District, Xi’an 710032, People’s Republic of China,

Tel: +86-015691759599

Email: liukun5959@qq.com

**Additional Editor Comments (if provided): Thank you for submitting your manuscript. Although it has been read with great interest, there are several aspects related to the methodology that are deserving special attention. For instance, aspects of the data set and the splitting data for training and validation are not clear, as it is the case with mismatching results and analysis.**

**Thank you for your valuable feedback. We have carefully considered your comments and have thoroughly revised the methodology and data sections of our manuscript to match with results and analysis.**

**<Methods>**
**Reviewer #1: None
Reviewer #2: The objectives of the study are clearly explained. I'd like to thank the authors for updating the methods sections to provide further details, but I would still like a bit of clarification on a couple of points:**

1. **Line 120: the data set referenced here doesn't list Ebola cases, rather it lists the health facilities in DRC. Please can you check this and provide the correct data reference.**

**Thank you very much for your valuable feedback and thorough review of our work. We deeply apologize for this mistake of incorrectly citing the dataset in the literature. We have addressed this problem, and the correction can be found in Pages 11-12, Lines 120-121. The correct dataset link is: https://data.humdata.org/dataset/ebola-cases-and-deaths-drc-north-kivu. In addition, we have uploaded both the raw and processed data to a public platform, anyone can access and download the data directly via link (https://www.researchgate.net/profile/Jiawei-Song-14/publication/386178115_Assessing_the_ecological_resilience_of_Ebola_virus_in_Africa_and_potential_influencing_factors_based_on_a_synthesized_model/data/6747e1be790d154bf9afacef/DataforManuscriptPLOSntd.zip?_tp=eyJjb250ZXh0Ijp7ImZpcnN0UGFnZSI6InB1YmxpY2F0aW9uIiwicGFnZSI6InB1YmxpY2F0aW9uIiwicG9zaXRpb24iOiJwYWdlQ29udGVudCJ9fQ).**

2. **Line 124: I don't think the description of HDX really adds anything here as you don't use data from over 250 countries and the resolution varies by data set.**

**Thank you for your valuable suggestion. We have removed the unnecessary description of the HDX platform to make the content more concise and to avoid any potential ambiguity (Page 12, Lines 121-124).**

3. **Line 160: I'm unclear about the purpose and method behind generating extra points via random sampling.**

**Thanks for your valuable comments. We understand your concern and here gives an explanation. Regarding the purpose of generating extra points via random sampling, from a Geographic Information Systems (GIS) perspective, randomly distributing points are considered as a more realistic representation of spatial data by avoiding over-concentration at a single location. Since our case data is available at the provincial level, directly mapping it to the provincial centroid may ignore the internal spatial distribution pattern within the province. By randomly generating points within a defined area can capture the heterogeneity of the spatial patterns and further reflect the spatial attributes of the variables. This also enhances the model's capability to represent the true distribution of Ebola cases and improves the accuracy of spatial analysis and predictions.**

**As for the method, we utilized the Random Point Generation tool in ArcGIS software to perform this operation. We specified the provincial administrative boundaries and the output point feature class, with the number of points determined based on the proportion of cases in each province relative to the total number of cases. This process resulted in the final output. To make it more clear, we have incorporated a description of the method in the manuscript (Page 13, Lines 158-162).**

4. **Line 209: You mention training the model. This would imply a training data set and a testing data set. Please can you provide further details of this?**

**Thanks for your professional concern which we really appreciate. We would like to give an explanation of this. In this study, the dataset was randomly partitioned into a training set (75% of the data) and a test set (25% of the data) for model validation. Each model was constructed using the training set and subsequently validated based on the test set. This information has been added into the manuscript (Page 16, Lines 224–226) for clarification. To assess the predictive performance of the models, we employed receiver-operating characteristic (ROC) curve analysis and calculated the area under the curve (AUC).**

5. **Figure 1: I think more thought needs to be put into this figure and what you want it to show. The time scale is kind of confusing, as well as the way that total case numbers are assigned to the month of outbreak in a country. It also gives the impression that Guinea, Liberia and Sierra Leone are separate outbreaks whereas they are all part of the same outbreak. Two separate outbreaks in DRC have been conflated (one was declared in May 2018, one in August 2018). I'm not sure that CFR (should be ratio rather than rate!) is helpful or relevant to the study. The CFR for the single case in Senegal (which is again, really part of the West Africa outbreak) should either be 0 or 1, not 0.4!**

**Thanks very much for pointing out this problem. The subfigure (a) is purposed to provide readers with a spatial and temporal perspective of the study area（Africa）and the whole distribution of selected EVD cases, making the information more clearly visualized. Subfigure (b) is used to show quantitative information of several significant outbreaks of Ebola virus disease, serving as a description of the historical EVD outbreaks for the subsequent analysis.**

**We really appreciate your comments and concern regarding the time scale on the x-axis, which indeed causes confusion. Thus, we have combined the year and region together to directly indicate which year and region experienced an outbreak. As the reviewer mentioned, the 2014 outbreaks in Guinea, Liberia, and Sierra Leone were part of the same epidemic. Here we differentiated them due to their distinct geographical contexts which may cause spatial heterogeneity. For the 2018 outbreaks in the DRC, although there were two separate outbreaks, we have combined them given their occurrence in the same year and region.**

**We also thank you for pointing out the problem with the case fatality ratio (CFR). According to the reviewer’s suggestion, we have corrected the label name from *rate* to *ratio* and revised the data accordingly. Through a careful review of our data sources, we identified some missing case data and errors in CFR calculations. We have updated the revised figure to ensure accuracy as shown below.**

6. **General: Thank you for providing more information on the data and the models (both in the text and Appendix B). However, for anyone hoping to repeat your analysis, I think it is still hard to understand what the models need in terms of data and what the model parameters are. I understand that the models that previously been published in Ecology journals (thank you for adding references), but they will likely be new to readers of PLOS NTDs. Given the data described so far, it is unclear to me how the input data requirements for the Maxent model (with influential factors x and species occurrence y) corresponds to the points A and B in p-dimensional space for the bioclim model? Do the models require the same or different data processing steps? How many parameters does each model have?**

**Thank you for your feedback and suggestions. We understand your concern and appreciate your insights, which help us further clarify the methodology. Below is the explanation to address these concerns,**

(1) **Data Requirements:**

**For all the four models in this study, we used the same primary datasets:**

a. **Species occurrence data: it is provided as a .txt file containing the spatial location (latitude and longitude coordinates) of each Ebola case.**
b. **Environmental variable data: it is provided in .tif format as continuous raster data.**

**While the input datasets are consistent across models, each model has specific requirements for data formatting. A summary of the input data requirements is shown in the following table to aid reproducibility.**

| **Model name** | **Data format of the case distribution point** | **Data format of the variables** |
| --- | --- | --- |
| Maxent | .txt | .asc |
| Boclim/Domain | .shp | .grid |
| GARP | .xls | .asc |

(2) **Input Data for Maxent and Bioclim Models:**

**Both Maxent and Bioclim models use species presence data and environmental variable data as input. For the Maxent model, species presence points are directly utilized to define the species' known distribution, while environmental variable data in continuous raster layers covering the entire study area, are used to extract environmental values at the species presence points. These extracted values serve as features to construct a probability distribution model. In contrast, the Bioclim model focuses on the range of environmental variable values (e.g., minimum and maximum temperatures) at species presence points, defining a multi-dimensional environmental space (p-dimensional, where p is the number of variables). Locations where the environmental conditions fall within the space are recognized suitable, with the model assigning a suitability score accordingly.**

**To address how the input data requirements of the Maxent model correspond to the points A and B in p-dimensional space for the Bioclim model, it is important to note both models use the same set of species presence and environmental variables. However, the mechanism how these two models utilize the environmental variables are different. For Maxent model, each species presence point is associated with a set of environmental variable values that serve as features to construct the model. Furthermore, the same set of environmental variable values correspond directly to the coordinates of the same species presence points in the p-dimensional space constructed by the Bioclim model. For Bioclim model, these points are used to define the boundaries of the species’ ecological niche, forming an environmental envelope. Thus, the linkage of two models are that same data that Maxent uses for feature extraction and probability estimation serve as the basis to identify the environmental boundary for Bioclim.**

(3) **Model Parameters:**
a. **Maxent Model: The required parameters are packed in the software are as follows:**
   a) **Feature classes (fc): LQ (Linear and Quadratic features).**
   b) **Regularization multiplier (rm): it controls the regularization strength with higher values yielding broader distributions, and normally it is assigned a value of 2.**
b. **Bioclim and Domain Models:**

**These models were executed using the default settings of the DIVA-GIS software. Readers can refer to section 7.1 of the DIVA-GIS user manual (https://diva-gis.org/docs/DIVA-GIS_manual_7.pdf) for parameter details.**

c. **GARP Model: The DesktopGARP 1.1.3 software was used with the following parameters:**
   a) **Runs = 20**
   b) **Convergence Limit = 0.01**
   c) **Max Iterations = 1000**
   d) **Models Under Hard Threshold = 20**
   e) **Commission Threshold = 50**

**A tutorial video on DesktopGARP parameter settings is available at https://www.youtube.com/watch?v=vIzRiXA2aOE.**

**We have updated the Appendix B to include a table that summarizes the input data requirements for each model and added more description to ensure greater clarity on these aspects, which can be found in highlighted color. Hopefully such revisions can make the methodology more transparent and reproducible for readers. Thank you again for your helpful comments!**

**<Methods>**
**Reviewer #1: None
Reviewer #2:**

7. **The results generally match the analysis plan. However, given that in the introduction, one of the main motivations for combining four distinct models was that relying on a single specific model may cause a decrease in accuracy due to inherent limitations, I would have expected to see some kind of analysis showing the benefit (or lack thereof) or synthesizing models as oppose to relying on one.**

**We appreciate your insightful comment. As discussed in the Methods section (Page 15, Lines 188-200) and more detailed in Appendix A, Table S1, we have examined the advantages of the ensemble model compared to each individual one. In addition, we have also incorporated the reference of Bonissone et al. (2011) into the Introduction section (Page 10, Lines 82-86) to further clarify the motivation in combining multiple models. Bonissone et al. (2011) emphasizes that *a fusion of these multiple models could generate an overall model with lower uncertainty and higher accuracy than is possible with a single model. A principal advantage of combining multiple models is the avoidance of loss of information that might occur if only the best performing model is selected.* Although the AUC yielded by the ensemble model that does not exceed that of the best-performing single model (e.g., Maxent), it can still mitigate the limitations of relying on a single or individual model. Its benefits extend to retain information beyond the accuracy metrics. The proposed weighted fusion approach makes use of the complementary strengths of each individual model, and thereby reduces uncertainty and mitigates the potential information loss. This trade-off aligns with our initial motivation to explore the combined model approach.**

8. **Line 302: What are the 100 experiments and how are they conducted? Did I miss something in the methods section?**

**Thank you very much for your valuable feedback. We sincerely apologize for the omission of this description in the Methods section. The 100 experiments refer to the significance analysis of the impact factors. Specifically, we performed numerous 10-fold cross-validations using the Maxent model for Ebola data, from which we selected 100 reasonable experimental results and averaged them. These results were used to calculate the contribution of each variable, permutation importance, and Jackknife test outcomes. Repeated experiments are essential for ensuring the robustness and reliability of the results, as they reduce the influence of random variations and provide a more comprehensive understanding of the model’s performance. We have revised the Methods section (Page 17, Lines 237-239) to added a detailed explanation of this process.**

9. **Line 327: How where the 1000 points generated?**

**Thank you for your insightful comment. We have moved the methodology regarding the generation of 1,000 random points from the Results section to the Methods section (Page 17, Lines 249–254) for better alignment with the structure of the manuscript. These points were generated using the Create Random Points tool in ArcGIS software, which ensures an even distribution across the study area while avoiding spatial bias. This approach provides representative environmental data, supporting a robust analysis of the associations between influencing factors and the Maxent model outcomes.**

10. **I also think the image quality needs to be improved (see Editorial and Data presentation modifications?)**

**Thanks for your very kind reminder. We also notice this problem and have checked through the resolution and readability of all figures, and we found the fuzzy figure might not be caused by the original uploaded figure. Before uploading all figures, we have checked each of them and make sure they are in the right format (TIF) and high resolution (exceeding 300 dpi). We guess this problem can be caused by creating the combined files in the submit system and appear fuzzy in the system-generated PDF. Reviewers might click the ‘Click here to access/download’ link in the top right corner of the PDF file, the high-quality images can be directly downloaded in the original resolution. We hope this can resolve any concern about the image quality.**

**<Conclusions>**
**Reviewer #1: None
Reviewer #2:**

12. **In the abstract and conclusions, the authors mention that their findings suggest new way to respond to small outbreaks, but nothing about this and public health is really discussed.**

**Thank you for pointing out this problem. The reviewer is correct and our original expression in the abstract was misleading. We have replaced the word *smaller* with another proper word from this sentence, and it is *Our findings suggest new ways to effectively respond to potential Ebola outbreaks in Sub-Saharan Africa.* (Page 6, Lines 23-24). Hope this change can avoid any confusion and misunderstanding.**

13. **The limitations focus on the limitations of data availability and don't really think about limitations of the models, what they can or can't do and what errors or biases different stages of the data processing might introduce.**

**Thanks very much for your professional and valuable comment. We have carefully addressed your concerns by incorporating a discussion from the perspective of the model itself in the limitations (Page 28, Lines 437-442). Specifically, we have elaborated on the limitations of our approach as follows:**

***Our synthesized model focuses on the static ecological adaptability and does not incorporate the spatial interaction dynamics, thereby limiting its capacity to simulate the mechanisms of Ebola virus spread across regions. Additionally, the framework lacks a hierarchical multi-scale analytical design, constraining its ability to disentangle complex interactions at varying spatial and temporal resolutions.***

**Regarding the potential errors or biases during data processing, comprehensively utilizing multi-source raster datasets in varying resolutions involved normalization and resampling. These operations may introduce uncertainties, such as the loss of fine-scale details or interpolation-induced artifacts, which could slightly affect the accuracy of subsequent analyses. We also added this point in the limitations (Page 28, Lines 442-448) as follows.**

***In addition, utilizing multi-source raster datasets at varying resolutions in one model involved normalization and resampling, and these steps may introduce uncertainties, such as the loss of fine-scale details or interpolation-induced artifacts, which could slightly affect the accuracy of subsequent analyses.***

14. **I also think the study is conflating two 'ecological' factors: one is where the zoonotic spillovers might occur, and one is where the disease might spread when the spillover has already occurred. I think this issue needs a more nuanced response.**

**Thank you for raising this important point. Our study focuses on constructing an ecological niche model to identify the suitable habitats for the Ebola virus based on the current distribution of cases and environmental factors. The primary objective is to assess the risk of potential outbreaks rather than the subsequent spread of the disease following a zoonotic spillover.**

**We acknowledge that exploring the disease spread post-spillover is a valuable research direction. However, to address this question requires establishing a more robust susceptible (S)-infectious (I)-recovered (R) model which needs more consideration of combing the physical process and neural network to reflect the dynamic mechanism and variable interpretability. Also, additional factors including the host behavior and population changes are needed. However, at current stages we cannot access to such data and just focus on identifying the suitable habitats for the Ebola virus. In further research, we have a plan to develop a new physical and deep learning model by incorporating more comprehensive datasets to research this challenging question. We sincerely appreciate your insightful suggestion, which does helps guide the direction of our next research work. For this concern, we have also discussed this in the limitation paragraph and the future direction of further study on Page 28, Lines 434-448.**

**<Editorial and data presentation>**
**Reviewer #1: None
Reviewer #2:**

15. **Many thanks to the authors for updating the figure numbers. However, the figure resolution is pretty low and it is hard to make out the names of the countries in figures 1, 4, and 5, and the scales in figures 7 and 8. These scales should also have units and y axis labels (at least for the leftmost figures.)**

**Thank you for your professional comment. Regarding figures, we would like to clarify that the original images provided in the manuscript are of sufficient resolution to clearly display the names of the countries and the scales. Before uploading all figures, we have checked each of them and make sure they are in the right format (TIF) and high resolution (exceeding 300 dpi). We guess this problem can be caused by creating the combined files in the submit system and appear fuzzy in the system-generated PDF. We kindly suggest readers to download the figures by clicking the top right corner of the PDF.**

**According to reviewer’s suggestion, we have revised Figures 7 and 8 to include complete labels for the y-axis and added units for all variables where applicable. The updated figures (compressed versions) are shown below. For Figure 8, which presents results from a generalized additive model (GAM), annotations are typically included to clarify the smoothing applied through the s() function. This notation is usually placed next to the y-axis. The second parameter within the s() function specifies the degree of smoothness, with smaller values producing a smoother curve and larger values resulting in more pronounced fluctuations.**

Figure 7. Response curves of environmental factors and the probability of suitability for Ebola based on Maxent model. Each curve (a-m) represents model-predicted probabilities associated with bio3, bio4, bio6, bio7, bio11, bio12, bio14, GPP, GPW, HII, LCCS, light, and NDVI. Curves display the mean response derived from 10 replicate Maxent runs (shown in red) with means and standard deviations (represented in blue, with two shades for categorical variables). **(revised)**

Figure 8. GAM model-based impact factors and ecological adaptability response of Ebola (a-m), final fitness classes with bio3, bio4, bio6, bio7, bio11, bio12, bio14, GPP, GPW, HII, LCCS, light, and NDVI. **(revised)**

16. **I'm grateful to the authors that they have included a key for the variable names in table 1. However, if would also be helpful to have the proper variable names in the figures and table 2 so that the reader doesn't keep have to referring back to table 1 in order to understand the results.**

**Thanks for your constructive comment. According to the reviewer’s suggestion, we have updated Table 2 (Page 22), as well as Figures 7 and 8, to include the full variable names. Hopefully these revisions can make it easier for readers to understand the results without having to refer back to Table 1. Your suggestion is extremely helpful to improve the quality of our manuscript and we really appreciate it. The revised table and figures are as follows.**

Table 2. Contribution and permutation importance of variables derived through Maxent modeling (average of 100 experiments). **(revised)**

| **Variable** | **Contribution (%)** | **Permutation Importance** |
| --- | --- | --- |
| bio4(Temperature Seasonality) | 44.89 | 48.19 |
| GPW(Gridded Population of the World) | 33.43 | 32.69 |
| bio12(Annual Precipitation) | 9.76 | 9.09 |
| LCCS(Land Cover Classification System) | 7.21 | 5.67 |
| bio6(Min Temperature of Coldest Month) | 2.47 | 2.82 |
| HII(Human Influence Index) | 1.55 | 0.59 |
| bio3(Isothermality) | 0.52 | 0.44 |
| GPP(Gross Primary Productivity) | 0.14 | 0.43 |
| NDVI(Normalized Difference Vegetation Index) | 0 | 0.06 |
| Light(Night Lights) | 0 | 0 |
| bio7(Temperature Annual Range) | 0 | 0 |
| bio14(Precipitation of Driest Month) | 0 | 0 |
| bio11(Mean Temperature of Coldest Quarter) | 0 | 0 |

Figure 7. Response curves of environmental factors and the probability of suitability for Ebola based on Maxent model. Each curve (a-m) represents model-predicted probabilities associated with bio3, bio4, bio6, bio7, bio11, bio12, bio14, GPP, GPW, HII, LCCS, light, and NDVI. Curves display the mean response derived from 10 replicate Maxent runs (shown in red) with means and standard deviations (represented in blue, with two shades for categorical variables). **(revised)**

Figure 8. GAM model-based impact factors and ecological adaptability response of Ebola (a-m), final fitness classes with bio3, bio4, bio6, bio7, bio11, bio12, bio14, GPP, GPW, HII, LCCS, light, and NDVI. **(revised)**

17. **For table 1, a couple of the data sources are missing, and I would like some clarity of the meaning and relevance of the means, maximums, minimums, etc. The area chosen for the study is Africa, but these values can't belong to Africa. Please can you check and amend these?**

**Thank you for your valuable feedback. We would like to clarify that all data sources for Table 1 are complete. The appearance of missing sources is due to the formatting of the three-line table. All variables in the table, including bio3, bio4, bio6, bio7, bio11, bio12, and bio14, are derived from the same source: the WorldClim database (https://www.worldclim.org/data/worldclim21.html).**

**According to your suggestion, we have added explanations of the means, maximums, and minimums below the table (Page 19, Table 1). For the values present not just for Africa, here is an explanation. Firstlly, Table 1 describes the characteristics of the selected variables based on the raw global dataset, and Africa is part of the global map and its values are within the threshold range of global dataset. So we think using to global dataset to give a description of the variables makes sense. Secondly, we agree with the reviewer that here using merely African values is more suitable. So we tried to cut Africa dataset from the whole global dataset, but it is difficult to make it since the global dataset is huge to be cut and be recalculated different values. So we have to give up this work and just keep the global dataset values. We think as long as it does not affect the relevance or validity of research, it is acceptable to use the global dataset to just give an introduction of those variables.**

**<Summary and General Comments>
Reviewer #1: None
Reviewer #2:**

18. **I think there is still some confusion over what the aim of this paper is and what it does that is not already in the literature. The authors claim that other studies do not take into account spatiotemporal trends in EBOV adaptability, whereas I believe some of them do. I think there is a bit of confusion over modelling zoonotic spillover, versus modelling human to human transmission that needs to be more carefully though about. The authors claim that synthesizing 4 ecological models provide a better understanding of Ebola's niche, but don't really demonstrate it. I suggest that they think a little bit more about where they want to position this paper within the literature and focus on achieving those aims first and foremost.**

**Thanks for your insightful concern which we really appreciate. We acknowledge that our original expression in the Introduction section was not clear. Our intention was to emphasize that some traditional mathematical models do not adequately capture the spatial variation of Ebola pathogens. However, we recognize that some previous studies indeed account for the spatiotemporal trends in EBOV adaptability, and our study is more about using multiple variables to extract the potential habitat of Ebola virus from a spatiotemporal perspective. Here we have changed the previous improper expressions to avoid any confusion (Page 10, Lines 82-86).**

**Regarding the focus of our study, our research specifically aims to identify suitable habitats for the Ebola virus and does not address the subsequent spread of the disease following zoonotic spillover events. This has been explained in our response to Comment 14 above. We also added it in the revised manuscript (Page 28, Lines 434-448), and we acknowledge that modeling human-to-human transmission will be an important direction for our future research.**

**Furthermore, as mentioned in our response to Comment 7, we integrated four ecological models using a weighted fusion approach to minimize the limitations of individual models and reduce potential information loss. While our study applies this integration specifically to Ebola, the concept of model integration has been noted in previous studies as a meaningful approach. We have added more explanation and reference support to demonstrate it in the text (Page 10, Lines 82-86). Since the primary aim of our study is to apply an ensemble modeling approach to predict the suitable habitats for the Ebola virus and to assess the potential risk. We analyzed multiple variables and the virus’s responses to the key environmental factors, and finally provide with ecological explanations for the observed patterns. These contributions can improve the understanding of the virus’s ecological niche from a spatiotemporal perspective and offer some valuable insights for future risk management. We sincerely thank you for your professional and valuable comments, which have greatly improved our work.**
